# Inferior alveolar nerve damage related to dental implant placement. A systematic review and meta-analysis

**DOI:** 10.4317/medoral.27125

**Published:** 2025-04-06

**Authors:** Juan Francisco Peña-Cardelles, Jovana Markovic, Samuel Akhondi, Ignacio Pedrinaci, Alejandro Lanis, German O Gallucci

**Affiliations:** 1 Department of Restorative Dentistry and Biomaterials Sciences, Harvard School of Dental Medicine, Boston, Massachusetts

## Abstract

**Background:**

A concern associated with implant placement is the potential occurrence of neurovascular lesions and subsequent development of sensory alterations in patients undergoing implant placements. The objective of this review is to evaluate the incidence of neurosensory alterations based on the proximity between the implant and the mandibular canal

**Material and Methods:**

A systematic review was conducted in MEDLINE, Web of Science, and Scopus. Studies with common variables were selected to conduct a meta-analysis. The patient classification was based on the mandibular canal-implant distance. Neurosensory alteration percentages were calculated for each study and group.

**Results:**

The findings indicate significant correlations between implant placement proximity and neurosensory risks. The incidence of neurosensory alterations in patients with implants placed at a distance ≥ 2 mm from the mandibular canal was 0%. Similarly, for distances between 1-2 mm from the mandibular canal, the incidence remained at 0%. However, for implants placed at a distance of 0-1 mm from the mandibular canal, the incidence of neurosensory alterations was 68%. Additionally, patients with implants that intruded into the canal had an incidence of 53% in the development of neurosensorial alterations.

**Conclusions:**

A distance of 1 mm from the mandibular canal might be safe. Implants placed at a distance less than 1 mm from the mandibular canal exhibit neurosensory alterations. Clinicians should be aware of the potential risk of nerve injury and adopt appropriate precautions, including meticulous preoperative planning and three-dimensional radiographic images.

** Key words:**Inferior alveolar nerve injury, mandibular canal, neurosensory disturbances, dental implant complications.

## Introduction

In recent years, noTable progress has been observed in the field of dental implantology, establishing dental implants as a reliable treatment modality for replacing missing teeth (1). Similar to other treatment modalities, implant dentistry is not exempt from complications. A major concern associated with implant placement is the potential occurrence of neurovascular lesions and subsequent development of sensory alterations in patients undergoing this treatment (2).

The prevalence of implant placements has experienced a noTable surge, which, in turn, has led to a corresponding increase in the documentation of neurovascular injuries over time in the scientific literature. Neurovascular complications tend to be most frequent in the mandibular region (2).

This complication may occur either from direct nerve trauma or as a consequence of indirect trauma, such as pressure exerted by a hematoma surrounding the neurovascular canal. Additionally, instances of chronic neuropathy have been reported in cases where implants were positioned close to the nerve without direct contact, leading to chronic stimulation (3).

The nervous system conFiguration within the mandible has been extensively investigated to identify risks and prevent injury to neural structures (3,4). Various types of nerve lesions are classified based on the extent of physical damage and associated symptomatology, which is crucial for prognostic considerations. These classifications include neuropraxia, axonotmesis, and neurotmesis (4).

The occurrence of neuropathic pain after implant therapy shows variations across different studies, ranging from 0% to 24% in the literature (3,5). Among the affected nerves, the inferior alveolar nerve (IAN) is the most commonly injured, followed by the lingual nerve. The primary factors contributing to IAN lesions are mainly iatrogenic, and the resolution of these injuries often takes more than 8 weeks. Nevertheless, approximately 80% of cases recover normal sensation within 6 months post-injury, with 91% experiencing full recovery after one year (6).

A safety distance of 2 mm was initially established by Misch and Crawford and later corroborated by Bartling *et al*. This distance has been widely accepted in the literature as a means to mitigate nerve damage and neurosensory alterations associated with implant placement. Although more accurate diagnostic modalities, such as cone-beam computed tomography (CBCT) appeared in this field, the 2 mm safety distance based on panoramic X-ray studies remains a standard reference (7,8).

The objective of this review is to evaluate the incidence of neurosensory alterations (NA) based on the proximity between the implant and the mandibular canal.

## Material and Methods

This systematic review was structured according to the Preferred Reporting Items for Systematic Reviews and Meta-Analyses (PRISMA®) Statement (9).

The study aimed to address the following PICO (*P*= patient/population/problem; I= intervention; C= comparison; O= outcome) question: "How does the distance (I) between the implant and the mandibular canal (C) impact the development of NA (O) in patients (P)?"

PICO components:

P: Patients underwent implant placements.

I: Distance between the implant and the mandibular canal.

C: Different distances between the implant and the mandibular canal.

O: Type of NA development.

-Inclusion Criteria:

The included articles met the following criteria: studies conducted on human subjects; randomized controlled trials; non-randomized controlled clinical trials; prospective or retrospective cohort studies, cross-sectional studies; case-control studies, case series with more than 20 cases, case reports, and observational studies. They should be published in English and published within the last 10 years.

-Exclusion Criteria:

The exclusion criteria were defined as follows: studies conducted on animals; investigations carried out on cadavers; experimental laboratory studies; systematic reviews and meta-analyses; duplicated publications; books or book chapters; letters to the Editor; and commentaries.

A thorough literature search was performed up to March 3rd, 2023 across the following databases: MEDLINE (via PubMed), Web of Science, and Scopus. Additionally, the bibliographic references of the selected articles were meticulously reviewed to identify relevant publications that did not appear in the initial search and might be of interest. Two independent researchers performed the search (JF. P-C. and J.M). MeSH (Medical Subjects Headings) terms, keywords, and other free terms were used with boolean operators (OR, NOT, AND) to combine searches: (Neuropathic OR nerve injury OR neurosensory disturbance OR numbness OR sensory disturbance OR dysesthesia OR paresthesia OR altered sensation) AND dental implant. The same keywords were used for all search platforms, following the syntax rules of each database.

-Study records

Two researchers (JF. P-C. and J.M). independently compared results to ensure completeness and removed duplicates. Then, the full title and abstracts of the remaining papers were screened individually. Finally, full-text articles included in this systematic review were selected according to the abovementioned criteria. Disagreements over which eligible studies were to be included were discussed with a third reviewer (AL) and a consensus was reached. Agreement between reviewers was measured with the Kappa coefficient. The results were also expressed as the concordance between reviewers (92,5%). If necessary, study authors were contacted for clarification or missing information.

Data were gathered from text and Tables. Before extraction, a calibration exercise was conducted to ensure consistency among reviewers, involving simultaneous data extraction from one eligible study. Any disagreements during this phase were addressed through discussion, and if both reviewers remained in disagreement, a third reviewer (A.L) was consulted for the final decision. Data on the following were extracted from the articles: identification of the study (authors, year of publication, and study design); sample characteristics (sample size, number of patients/number of implants, frequency of neurosensory disturbances, type of neurosensory alterations, radiographic method used, implant distance to the mandibular canal, time to recovery).

-Meta-analysis

Those studies with common variables and homogeneity were selected to assess the possibility of performing a meta-analysis. Studies should register the number of implants, number of patients, and inferior nerve canal distance from the implant and register NA. Studies with common variables and homogeneity were selected to conduct a meta-analysis. The selected studies were required to report the number of implants, number of patients, and registered distance between the implant and the NA.

Patient classification was based on the nerve canal-implant distance and grouped as follows: 0-1 mm of distance, 1-2 mm of distance, and ≥2 mm of distance.

Neurosensory alteration (NA) percentages were calculated for each study and group, categorized as either hyperesthesia and/or neuropathic pain group or hypoesthesia group, following the definitions provided by the International Association for the Study of Pain (IASP).

A quantitative synthesis using a meta-analysis software program was performed (SPSS v28, IBM SPSS, 2021). Fixed- or random-effect models were applied based on the heterogeneity among studies. The forest plot was used to illustrate the weighted mean of the outcome in each study and the final estimate.

-Risk of bias in individual studies

The authors assessed the included studies' quality and risk for bias by the Newcastle-Ottawa Scale (10). The selected studies were observational studies, for which this bias assessment protocol is specifically appropriate. This was done independently and in duplicate by two authors (J.M. and S.A.). Any disagreement was discussed between the two authors and a third researcher was consulted when agreement did not exist (J.F.P.C).

## Results

-Study selection

**Figure 1:**
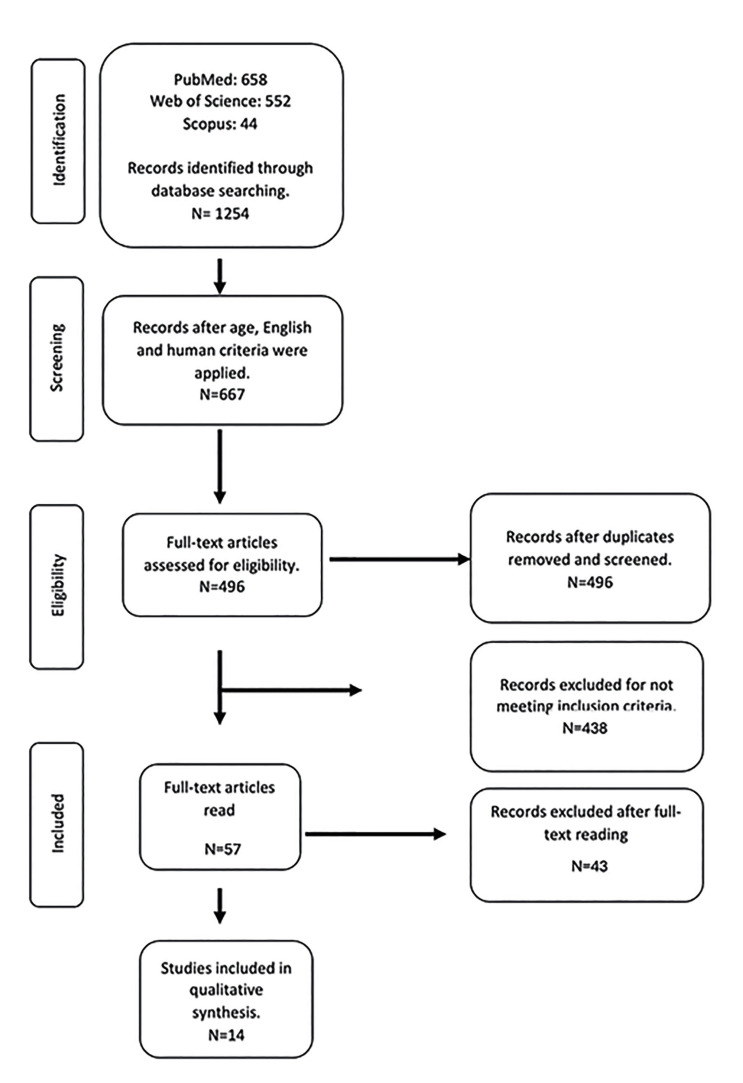
PRISMA® flow diagram of the search.

The search strategy yielded 1254 results (PubMed: 658, Scopus: 44, Web of Science: 552). After applying a chronological limitation of 10 years, 771 articles remained. Further application of the English language criterion reduced the count to 761 articles. Subsequently, by considering studies conducted on human subjects, the number was further reduced to 667 articles. After removing duplicates (*n*=171), 496 articles were retained. Two independent researchers (J.M. and J.F. P-C.) reviewed the titles and abstracts of the remaining articles, excluding 437 papers that fell outside the scope of this review, resulting in 58 potential references. Upon examining the full text of these 58 articles, 44 were excluded for not meeting the inclusion criteria. As a result, 14 observational studies were ultimately included in this systematic review (Fig. 1) (11-24). The excluded articles and reasons are represented in the Supplement 1. Characteristics of the included articles are represented in Table 1.

-Neurosensory Alterations and Sensory Testing

The studies reviewed focused on assessing sensory disturbances associated with dental implant placement, particularly concerning the proximity to the mandibular canal and other contributing factors (25). The findings indicate significant correlations between implant placement proximity and neurosensory risks, demonstrating the need for precise surgical planning and execution (5,11,13). One study assessed extra and intraoral NA of the IAN in 33 patients with dental implants on one side of the posterior region of the mandible. Sensory testing of the mental nerve innervation areas revealed no significant differences, although the mucosal lower lip showed lower sensitivity to touch, and pain compared to the chin. The average distance from the implant to the mandibular canal was 2.65±1.75 mm (11).

A retrospective study involving 34 patients diagnosed with trigeminal neuropathy following implant placement analyzed data from the Neuropathic Pain Symptom Inventory (NPSI), thermal and electric Quantitative Sensory Testing (QST), Qualitative Sensory Testing (QualST), and CBCT. Numbness was the most common symptom, observed in 91% of patients, while evoked pain was reported by 94% (12).

Further retrospective studies assessed the risk of NA in patients with implants placed within 2 mm of the mandibular canal in the posterior region. One study found that 1.3% of implants were positioned within 2 mm of the mandibular canal, whilst 0.39% were placed at a distance of less than 1mm from the mandibular canal. One implant placed within 0 to 0.99 mm of the mandibular canal presented with temporary NA (13). Another study involving 60 patients with 101 implants found that implants that resulted in NA had an average implant penetration into the mandibular canal of -0.86 ±0.5 mm (20).

Additional research corroborated the transient nature of NA and emphasized the importance of implant proximity to the mandibular canal, reporting transient alterations in 348 patients with implants placed close to the mandibular canal. NA were more prevalent when implants were placed within 1.5 mm of the canal (19). Another study identified a high incidence of implants affecting the accessory lingual canals, leading to NA in 27% of patients (19).

Another study examining 7,602 patients found that neurosensory damage to the trigeminal nerve branch occurred in 56 cases, with implant placement being a common cause. Hypoesthesia was the most frequent symptom, followed by neuropathic pain (24).

An analysis of 16 patients who all experienced sensory disturbances post-dental implant placements identified hyperalgesia as the cause in 31.25% of cases and hypoalgesia in 68.75%. Postoperative sensory disturbances were often related to bleeding during surgery, pain during drilling, and modifications to the initially chosen implant size. This study found that dental implants were the most common etiological factor for nerve injury (22).

-Demographic and Clinical Factors Affecting Implant Success

Demographic factors such as age and gender impact the risk of nerve damage during implant procedures. One study observed that older women are more susceptible to IAN damage, suggesting the use of a CBCT to examine nerve distribution, due to inappropriate radiographic examinations being a common cause of nerve damage (14). Another retrospective cohort study determined the prevalence of neuropathic pain and NA after dental implant placement, highlighting that most cases occurred in patients over 60 years old, primarily women (17).

An analysis of 1,065 patients receiving 3,025 implants found that while a significant percentage experienced sensitivity disturbances shortly after implantation, these typically resolved within 13 months. The study did not find a correlation between the bone above the mandibular canal, implant length, operator experience, or ridge atrophy and the incidence of altered sensation. However, it was noted that many cases of mandibular canal and IAN injury were evaluated only with dental panoramic X-rays, with few using CT or CBCT preoperatively (15).

-Management and Outcomes of Neuropathic Pain after implant placement

Of the studies exploring factors contributing to neuropathic pain following dental implant placement, one case series report observed 26 patients with neuropathic pain, noting that those who had implant removal within three months experienced symptom improvement. In contrast, patients who had implant removal after four months did not see their symptoms improve (16).

A retrospective cohort study examining 53 cases of iatrogenic trigeminal nerve injury found that dental implant placement was a common cause of nerve damage, often accompanied by pain. All cases with implant placement had persistent symptoms three months post-treatment onset. The study highlighted that third molar extractions were the most common cause of nerve injury, followed by implant placements (18).

-Preoperative Planning and Etiological Background

Radiographic imaging has been considered an important factor during the planning for dental implant procedures as it provides essential diagnostic information. Moreover, implementing adequate radiographic imaging techniques can lead to successful treatment outcomes. Thus, it is important to address which radiographic methods have been used in the included studies. Advanced imaging techniques, such as CT and CBCT have become a valuable tool for diagnosis. In most of the included studies, CBCT and CT techniques were utilized either in preoperative planning and postoperative diagnostics of neuropathic alterations (11,12,15,16-18,20,22,24). However, in some of the studies, Panoramic X-ray was used as part of preoperative planning and afterwards, postoperative diagnostics either in combination with CBCT and CT was used as only radiographic method (13,21).

A study involving 92 patients with neurosensory deficiencies related to dental implant placement revealed that the majority of cases were associated with preoperative planning relying on panoramic or periapical radiographs, rather than CT scans (23).

Despite CBCT imaging is a “gold standard” diagnostic tool, it has several limitations that need to be addressed: radiation exposure, artefacts and image distortions, especially when metallic objects are present, limited soft tissue visualization, lack of resolution capture for assessing thin bony structures, accuracy-related to measurements (patient movement, artefacts, voxel size) and radiation scattering and beam hardening artefacts that can affect image quality and disable visualization of areas with anatomical structures. (26-28).

The selected studies for meta-analysis and their classification in groups are described in Table 2.

-NA rate based on implant distance to the IAN canal.

**Figure 2:**
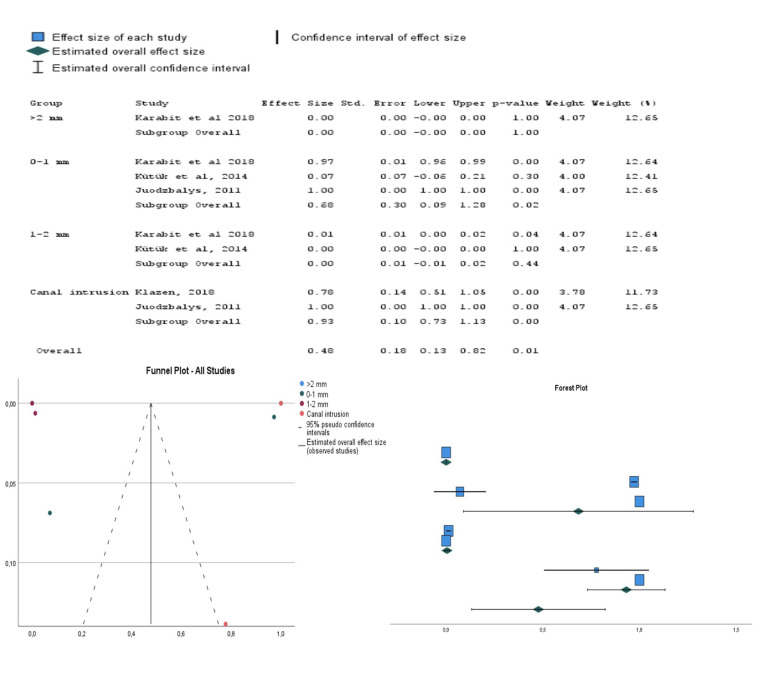
Meta-analysis of the NA rate based on implant distance to the mandibular canal.

The incidence of NA in patients with dental implants placed at a distance ≥ 2 mm from the mandibular canal was 0% (confidence interval: 0.00-0.00). Similarly, for distances between 1-2 mm from the mandibular canal, the incidence remained at 0% (confidence interval: 0.01-0.02). However, for implants placed at a distance of 0-1 mm from the mandibular canal, the incidence of NA was 68% (confidence interval: 0.09-1.28). Additionally, patients with implants that intruded into the mandibular canal had an incidence of 53% (confidence interval: 0.13-0.82) in the development of NA. (View Fig. 2).

-NA type based on implant distance to the mandibular canal.

-Hypoesthesia

**Figure 3:**
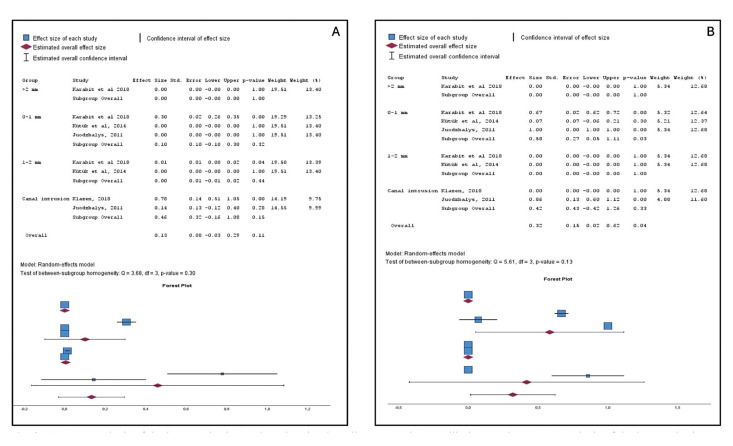
A. Meta-analysis of the hyperesthesia rate based on implant distance to the mandibular canal. B. Meta-analysis of the hypoesthesia rate based on implant distance to the mandibular canal.

Patients with implants placed at a distance of ≥ 2 mm from the mandibular canal and those in the 1-2 mm group exhibited a 0% incidence of NA (confidence interval: 0-0). However, patients with implants positioned between 0-1 mm from the mandibular canal had a NA development rate of 58% (confidence interval: 0.06-1.11). Furthermore, patients with implants that intruded into the mandibular canal presented hypoesthesia in 42% of cases (confidence interval: 0.42-1.26). Notably, both the 0-1 mm and mandibular canal intrusion groups showed significant differences concerning the 1-2 mm and ≥ 2 mm distance groups. (View Fig. 3).

-Hyperesthesia

**Figure 4:**
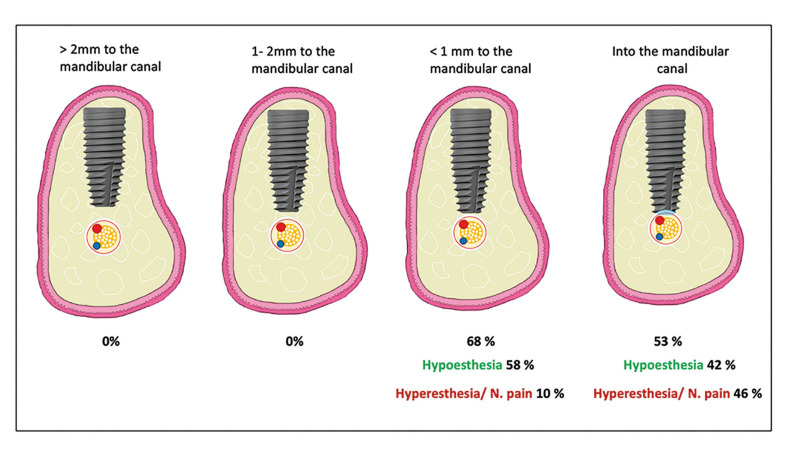
Incidence of NA based on nerve canal-implant distance.

The ≥ 2mm and 1-2 mm groups had hyperesthesia at a rate of 0% of the cases (with a confidence interval of 0-0 and 0.01-0.02 respectively). (View Fig. 4). Those patients in the 0-1 mm and mandibular canal intrusion groups had NA with hyperesthesia in 10% of the cases (confidence interval 0.1-0.3) and 46% (confidence interval 0.16-1.08) respectively. (View Fig. 3).

Outcomes related to the meta-analysis are illustrated in Fig. 4.

-Risk of bias in individual studies

Based on the Newcastle-Ottawa Scale assessment, the quality of studies varied. Five studies were classified as good quality due to high scores across all domains, indicating strong methodological rigor (12-15,19,20). Medium-quality studies showed minor limitations, typically in cohort comparability or follow-up adequacy (9,17,21,22). The remaining studies were rated as low quality due to lower scores, primarily in areas of follow-up length and cohort selection, affecting their reliability.10,11,16,18 The outcomes of the risk bias assessment are represented in Table 3.

## Discussion

Nerve injury is a recognized complication associated with dental implant placement in the mandible. The reported prevalence of such injuries exhibits substantial variation across studies. Some studies indicate low rates, with less than 1%, while others report higher Figures, ranging from 24% (29) and even one study identifying 43.5% nerve injuries following dental implant placement (30). This considerable diversity in reported percentages may stem from differences in receptor site anatomical conditions, surgical techniques (free hand vs guided surgery), utilization of digital planning, and variations in follow-up time.

Digital implant planning (from CBCT site evaluation to comprehensive virtual planning in dedicated implant software) should be regarded as the gold standard in contemporary dental implant dentistry. This approach allows for a detailed assessment of the anatomical conditions at the surgical site, ensures compliance with the site phenotype requirements for successful implant placement, identification of anatomical structures (e.g mandibular canal) and preventing potential complications or co-morbidities associated with the surgery (20,31,32).

However, in daily practice, different radiological techniques have been used for the assessment of the anatomical structures before implant placement procedures (CBCT, CT, Panoramic X-rays, and Magnetic resonance imaging). Even though CT has been a useful tool for digital implant planning and diagnosis, its high radiation dose puts CBCT as the preferable radiographic imaging in modern implantology. Panoramic x-rays are still being used as a diagnostic tool and they can provide a general overview of the upper and lower jaw with a small dose of radiation (13). However, panoramic-X-rays may also have limitations such as a degree of magnification of around 20-30% that can cause picture distortion, and inability to identify important anatomical landmarks accurately such as the mandibular canal (30). Most of the included studies in this systematic review and meta-analysis utilized CBCT or CT imagining in preoperative planning and postoperative nerve injury evaluation or a combination of 3D imaging with panoramic X-rays. However, two studies did not specify which radiographic imaging method was used (14,19) and only one study using a standardized measurement protocol in panoramic X-rays was included (21).

To enhance the systematic review's quality, we exclusively included studies published within the last ten years, ensuring broader access to digital planning tools for researchers. Consequently, the data reported in these studies originate from articles wherein researchers had access to virtual planning and post-operative CBCT scans, enabling accurate identification of the mandibular canal and measurement of the distance between the implant apex and mandibular canal. To emphasize the pivotal role of digital planning in mitigating nerve damage, a study included in our analysis, revealed that 76% of cases with NA relied on treatment planning with periapical or panoramic radiographs instead of utilizing virtual planning (23).

Not only digital planning is important but also the surgical technique (i.e guided vs free hand drilling and implant placement), or bone quality at the recipient site related to implant-related complications and nerve injuries (33,34). A study demonstrated, despite CBCT-based planning, IAN injury occurred due to mandibular canal intrusion of 0.86 ± 0.5 mm.20 Hence, while digital planning holds importance, the translation of the virtual plan into a clinical scenario through computer-assisted implant surgery (CAIS) becomes crucial to prevent potential deviations from the original plan and mitigate risks associated with nerve injuries (34).

Distance between the implant apex and the mandibular canal emerges as a critical factor influencing the risk of nerve injury. Studies consistently report that a smaller distance correlates with a higher risk of nerve injury. Consequently, implant design, 3D position during planning and mean deviation of the final implant position compared to the planned one should be considered during implant planning and placement. Particularly when employing free-hand surgical strategies (13,21,23).

The safety distance of 2 mm was confirmed in studies of Mish and Crawford (1990); and Bartling *et al*. (1999) seems rational, and has been well accepted in the literature until today (7,8). However, this distance was described a long time ago and Implant Dentistry has made advances with new implant surfaces, increased usage of narrow and short implants as well as computer-guided surgery. These evolutions represent a new era of modern implantology.

Bartling *et al*. (1999) included in this study a sample of 94 patients of which eight presented NA (7). The statistical results of the present meta-analysis have been extracted from 1636 patients who have received implants between the mandibular canal and 3 mm. Therefore, we consider that the same behavior of >1mm and >2mm regarding the development of NA found in this meta-analysis can apport a piece of updated relevant information about the safety implant distance topic.

A study reported NA in those patients with implants placed within the range of 0.5-1.5 mm from the mandibular canal.21 In contrast, another retrospective study of 1957 patients, reported only one case of temporary hypoesthesia when the implant was placed less than 2 mm from the IAN (11). The statistical findings of our meta-analysis, involving 1636 patients with distances between the apex of the implants and the mandibular canal of 1, 2 and 3 mm contribute updated and relevant insights to the safety implant distance dilemma.

Although prevention of IAN damage is advocated in this study, data reported in this systematic review yields a large variety of complications related to implant surgery, being permanent or temporary is considered one of the most serious complications clinicians could face (33,35). In a study, it was noted that after one month of implant placement 23 patients out of 1065 presented with sensitivity disturbance but all of them recovered after 13 months (13). One study reported the most common symptom was numbness in 91% (12), while some studies also reported frozen sensations, paresthesia, and neuropathic pain (16-18,21,23). The findings of two studies suggested that nerve injury is more common in women than in the male population. One of them found that women older than 60 years had trigeminal neuropathy as a consequence of implant placement (17). Nevertheless, the other one found in their study that women are 3.29 times more likely to get IAN injuries than male (14).

Caution must be exercised in interpreting this study’s results, given the heterogeneity and retrospective nature of the included studies. Nonetheless, strict inclusion/exclusion criteria were implemented to mitigate this limitation. These criteria and a time frame restricting articles to those published within the last decade resulted in studies evaluating mandibular canal-implant distance digitally using CBCT, except for only one study. Another challenge arises from variations in criteria defining nerve injury or reporting sensory disturbances across studies, limiting its comparability. Despite these limitations, meta-analyses were conducted only with a reduced number of studies, allowing for meaningful inferences.

The clinical implications of our findings suggest that maintaining a 1 mm distance between the implant apex and the mandibular canal may be sufficient to prevent the development of NA. When implants are placed within 1 mm of the canal, hypoesthesia occurs in approximately 58% of cases. However, when the implant penetrates the canal, hypoesthesia develops in 42% of cases; this injury may also result in hyperesthesia and neuropathic pain in 46% of cases. This phenomenon can be explained by the significant damage occurring within the mandibular canal, including fractures of the canal roof, internal hemorrhage, and bone debris deposits. These events are considered direct damage to the nerve, producing direct mechanical stimulation of nerve fibers, as opposed to indirect stimulation that may occur when implants are placed further away from the canal (36,37).

## Conclusions

Clinicians should be aware of the potential risk of nerve injury and adopt appropriate precautions, including meticulous preoperative planning and three-dimensional radiographic images since the incidence of nerve injury may be influenced by factors such as implant length, diameter, and the distance from the implant to the mandibular canal. This study concludes that a distance of 1 mm from the mandibular canal might be safe. Due to the limitations identified in this review, further research including prospective randomized clinical trials should be performed to confirm these findings.

## Figures and Tables

**Table 1:** Characteristics of included studies.

Author	Global sample size	Number of patients or implants studied with issues	Frequency of neurosensory disturbance	Radiographic method	Injury to the nerve during drilling	Distance to the mandibular canal	Implant removal as treatment	Type of neurosensory alteration/pain	Time to recover
Scarano et al. (15) 2017	3025 Implants3025 Implants	62 Implants	100%	CT or CBCT	NS	< 1mm	No	NS	1 month +-0.3
		12 Implants	100%	CT or CBCT	NS	Contact without intrusion	Yes	NS	1,5 month +-0.3
		8 Implants	100%	CT or CBCT	NS	Canal intrusion	Yes	NS	6 months +-0.5
Karabit et al. (21) 2018	1571 Patients,2432 Implants	560 Implants.	100%	Panoramic X-ray	-	< 1 mm	NS	Hypoesthesia and hyperesthesia	-
		121 Implants	100%	Panoramic X-ray	-	(0-0.45mm)	-	-	121 more than 12
		216 Implants	100%	Panoramic X-ray	-	(0.46-0.99mm)	-	-	216 6 to 12
		223 Implants	-		-	(0.76- 1 mm)	-	-	223 less than 1 week
		490 Implants	4 cases with alteration	Panoramic X-ray	-	1-1,99 mm	NS	Frozen sensation	4 Less than 1 week
		1382 Implants	0%	Panoramic X-ray	-	>2mm	NS	None	None
Froum et al (20) 2021	60 Patients,101 Implants	5 Patients / 9 Implants	NS	CBCT	YES	Canal intrusion	NS	Paresthesia (4) and neuralgia (1)	NS
		55 Patients/92 Implants	NS	CBCT	YES	< 2mm (0.14 to 1.8 mm) without contact	NS	None	NS
Pääsky et al. (14) 2022	178 Patients/405 Implants	178 patients/405 implants	NS	NS	NS	NS	NS	NS	NS
Chaar et al. (19) 2022	50 Patients(NS Implants)	50 Patients	NS	NS	YES	2mm-44 patientsAway from the canal-6 patients	NS	NS	3 months
Vazquez-Delgado et al (15) 2018	1012 Patients3743 Implants	8 Patients(NS Implants)	NS	Panoramic X-ray and CBCT	YES	Inside canal	NS	Pain, hypoesthesia, alodynia,Trigeminal neuropathy without pain	NS
Politis et al. (16) 2017	26 Patients	13 Implants	NS	Panoramic X-ray and CBCT	YES	NS	NS	Frozen sensations, paresthesia, and neuropathic pain	NS
Kütük et al. (13) 2014	1957 patients3608 Implants	34 Implants	NS	Panoramic X-ray and CBCT	NS	34 implants1-2 mm	One case	None	16 months
						14 implants 0-1mm		Neurovascular alteration	
Hartmaan et al. (11) 2016	33 Patients	33 Patients	NS	Panoramic X-ray and CBCT	YES	2.65 +-1.75 mm	NS	Numbness, temperature algesia	NS
		(NS Implants)							
Kim et al. (12) 2019	34 Patients	17 Implants	100%	CBCT and Panoramic X-ray	NS	No contact between the nerve and the implant	NS	Numbness, paresthesia/dysesthesia, burning pain	NS
		1 implant	100%	CBCT and Panoramic X-ray	NS	Contact without intrusion	NS	Numbness, paresthesia/dysesthesia, burning pain	NS
		14 Implants	100%	CBCT and Panoramic X-ray	NS	Partial encroachment	NS	Numbness, paresthesia/dysesthesia, burning pain	NS
		2 Implants	100%	CBCT and Panoramic X-ray	NS	Perforation of the IAN	NS	Numbness, paresthesia/dysesthesia, burning pain	NS
Klazen et al. (18) 2018	53 Patients	9 Implants	NS	CBCT	NS	Nerve injury inside	NS	Hyperalgesia	3 months
Juodzbalys et al. (22) 2013	16 Patients	1 Implant	NS	Axial CT-Scans	NS	Close to the canal without contact	NS	NS	5 days
		7 Implants	NS	Axial CT-Scans	NS	Into the canal	NS	NS	14h-52h
		3 Implants	NS	Axial CT-Scans	NS	Damage during drilling	NS	NS	13h-28h
Givol et al. (23) 2013	92 Patients92 Implants	4 Implants	0%	CT and Panoramic X-ray	No	2mm or more	No	NoneParesthesia	N/A
		1 Implant	100%	CT and Panoramic X-ray	Yes	1 to 2mm	Yes	Paresthesia, Dysesthesia	NS
		8 Implants	100%	CT and Panoramic X-ray	Yes	0mm	Yes	Hyperesthesia, Allodynia,	No Recovery
		28 Implants	100%	CT and Panoramic X-ray	Yes	Into the canal	Yes	Hyperalgesia, Paresthesia, Dysesthesia, Anesthesia, Hypoesthesia	No Recovery
Agbaje et al. (24) 2015	7602 Patients	56 Patients56 Patients (NS implants)	NS	Panoramic X-RayCephalometric radiographCBCTMRICT	NS	NS	NS	Hypoesthesia, neuropathic pain	NS

NS: No specified.

**Table 2:** The meta-analysis included articles.

Auhtor	Year	Number of implants	Distance to the mandibular canal	Neurosensorial alteration duration	Groups	Number of patients	Type of neurosensorial alteration
Karabit *et al*. (21)	2018	121 implants	0- 0.45 mm	More than 12 months	Group of 0-1 mm	361 patients	241 patients hypoesthesia (66.76%)
		216 implants	0.46- 0.75 mm	6 to 12 months			110 patients hyperesthesia (30.47%)
		223 implants	0.76- 1 mm	Less than 1 week			
		4 implants	1-1.99 mm	Less than 1 week	Group of 1-2 mm	316 patients	4 hyperesthesia (1.27%)
		1382	>2mm	None	Group >2mm	894	None (0%)
Kütük *et al*. (13)	2014	34 implants	1-2 mm	None	Group 1-2mm	34 patients	None (0%)
		14 implants	0-1mm	16 months	Group 0-1 mm	14 patients	1 hypoesthesia (7.14%)
Klazen *et al*. (18)	2018	9 implants	Canal intrusion	3 months	Group canal intrusion	9 patients	7 hyperalgesia (77,7%)
Juodzbalys *et al*. (22)	2013	7 implants	Canal intrusion	Less than 1 week	Group of canal intrusion	7 patients	6 hypoesthesia (85,71%)
							1 hyperesthesia (14,29%)
		1 implant	0-1 mm	Less than 1 week	Group of 0-1mm	1 patient	1 hypoesthesia (100%)

**Table 3:** Risk Bias Assessment.

Study	Selection				Comparability	Outcome			Total score
	Representiveness of the exposed cohort（1)	Selection of the non-exposed cohort（1)	Ascertainment of exposure（1)	Demonstration that outcome of interest was not present at start of study（1）	Comparability of cohorts on the basis of the design or analysis（2)	Assessment of outcome（1）	Was follow up long enough for outcomes to occur（1)	Adequacy of follow up of cohorts（1)	
Scarano *et al*. (2017) (15)	*	*	*	*	**	*	*	*	Good Quality
Karabit *et al*. (2018) (21)	*	*	*	*	*	*	*	*	Good Quality
Froum *et al*. (2021) (20)	*	*	*	*	*	*	-	-	Low Quality
Pääsky *et al*. (2022) (14)	*	*	*	*	*	*	*	*	Good Quality
Chaar *et al*. (2022) (19)	*	*	*	*	*	*	-	-	Medium quality
Vazquez-Delgado *et al*. (2018) (17)	*	*	*	*	**	*	*	*	Good quality
Politis *et al*. (2017) (18)	*	*	*	*	**	*	*	*	Good quality
Hartmaan *et al*. (2016) (11)	*	*	*	*	**	*	-	-	Medium quality
Kim *et al*. (2019) (12)	*	*	*	-	**	*	-	-	Low quality
Kütük *et al*. (2014) (13)	*	*	*	-	*	*	*	-	Low quality
Klazen *et al*. (2018) (18)	*	-	*	-	*	*	*	-	Low quality
Juodzbalys *et al*. (2013) (22)	*	*	*	*	**	*	*	*	Good quality
Givol *et al*. (2013) (23)	*	-	*	*	*	*	*	-	Medium quality
Agbaje *et al*. (2015) (24)	*	-	*	*	*	*	*	*	Medium quality
